# Impact of the ICU No-Visitation Policy on Delirium in Long-Term Mechanically Ventilated Patients: A Single-Center Retrospective Observational Study

**DOI:** 10.7759/cureus.89771

**Published:** 2025-08-11

**Authors:** Tomoo Sato, Kenta Ogushi, Takakazu Kitatsuji, Kana Mitsui, Yoshihiro Kageoka, Kaoru Makino, Hiroto Hanamachi, Yoshiki Sowa, Yukie Akamatsu, Kuniyasu Kamiya

**Affiliations:** 1 Division of Acute Care Nursing, Kobe City College of Nursing, Kobe, JPN; 2 Department of Nursing, Kobe University Hospital, Kobe, JPN; 3 Division of Nursing Theory and Practice, Kobe City College of Nursing, Kobe, JPN; 4 Division of Health Science, Kobe City College of Nursing, Kobe, JPN

**Keywords:** critical care, delirium, intensive care units, mechanical ventilation, visitation

## Abstract

Introduction

This study aimed to evaluate the impact of the no-visitation policy and the presence or absence of remote visitation during the policy period on delirium in intensive care unit (ICU) patients requiring mechanical ventilation and to obtain implications for critical care nursing practice. This research is important for informing ICU family visitation policies, as delirium is associated with increased mortality and prolonged ICU stay, and understanding the impact of family presence on delirium prevention has significant clinical implications.

Methods

This single-center retrospective observational study included patients who received mechanical ventilation for at least 48 hours between February 2019 and October 2022. Patients were divided into two groups based on before and after the implementation of the no-visitation policy due to the COVID-19 pandemic, and the incidence and duration of delirium were compared. Delirium was assessed using the Intensive Care Delirium Screening Checklist (ICDSC), and logistic regression analysis was used to analyze the impact of the no-visitation policy on delirium occurrence.

Results

The study included 359 patients (pre-policy group: 183 patients, post-policy group: 176 patients). Patient demographics included a median age of 69 years (interquartile range: 56-78), Acute Physiology and Chronic Health Disease Classification System II (APACHE II) score of 21 (range: 16-27), and mechanical ventilation duration of eight days (range: 5-17). The incidence of delirium was 72.7% (133/183) in the pre-policy group and 65.9% (116/176) in the no-visitation policy group (p=0.164). The duration of delirium was four (range: two to seven) days in both groups (p=0.593). There was also no significant difference in delirium incidence based on whether remote visitation was provided in the no-visitation policy period (p=0.81).

Conclusions

The no-visitation policy was not significantly associated with the incidence or duration of delirium in ICU patients requiring long-term mechanical ventilation. We are suggesting that comprehensive non-pharmacological interventions, rather than family visitation alone, may be essential for delirium prevention in high-risk ICU populations.

## Introduction

Delirium is a significant challenge in critical care, characterized by acute disturbance of consciousness with inattention and cognitive changes [1]. The incidence of delirium in intensive care unit (ICU) patients ranges from 30% to 80%, with particularly high rates in mechanically ventilated patients [2,3]. Delirium increases mortality, prolongs ICU and hospital stays, raises healthcare costs, and contributes to long-term cognitive impairment [4,5]. During the COVID-19 pandemic, delirium incidence exceeded a reported 50% in mechanically ventilated COVID-19 patients [6] and was identified as an important mortality predictor [7], highlighting the need for effective prevention and management strategies in ICU nursing practice.

Traditional pharmacological approaches to delirium have shown limited effectiveness and potential adverse effects. Antipsychotics have been reported ineffective in altering the course [8,9], with potential safety concerns [10]. Excessive sedative use carries risks of prolonged hospitalization and cognitive decline [11]. Given these limitations, current guidelines emphasize non-pharmacological interventions, specifically recommending family presence and participation as key elements in delirium prevention [12,13]. Evidence supporting the positive impact of family presence on ICU patients continues to accumulate. Fumagalli et al. [14] reported that flexible visitation policies contribute to reduced anxiety and psychological stability in patients, while a meta-analysis by Junior et al. [15] demonstrated that relaxed visitation restrictions were associated with reduced delirium incidence. Family presence is thought to function as a non-pharmacological delirium prevention by helping patients maintain reality orientation, providing reassurance, and offering opportunities for cognitive stimulation.

The COVID-19 pandemic necessitated no-visitation policies in ICUs worldwide, creating an opportunity to reassess the role of family in delirium prevention. Alternative approaches using digital technology, such as remote visitation via video calls, were implemented, though evidence regarding their effectiveness remains limited. The ICU Visits randomized clinical trial by Rosa et al. [16] reported that flexible visitation policies did not significantly impact delirium incidence, suggesting that this relationship requires further investigation. Few studies have examined the association between COVID-19 pandemic no-visitation policies and delirium incidence, particularly in high-risk patients requiring long-term mechanical ventilation.

This study aimed to evaluate the impact of no-visitation policies on delirium incidence and duration in ICU patients receiving mechanical ventilation for ≥ 48 hours and assessed whether remote visitation affected delirium outcomes during the restricted visitation period.

## Materials and methods

Study design and setting

This single-center retrospective observational study used a before-after comparative design with the implementation of the COVID-19 no-visitation policy (March 2020) as the boundary point. The study was conducted in a 20-bed adult ICU of Kobe University Hospital, Kobe, Japan, from February 2019 to October 2022. The ICU is a mixed medical-surgical ICU primarily serving patients with respiratory failure, sepsis, and post-operative complications. Before the pandemic, standard policy allowed one 30-minute visit per day (between 7:00-8:00 AM and 11:00 AM-8:00 PM). The no-visitation policy was implemented in March 2020 and continued throughout the study period, with remote visitation (video calls) offered to some patients as an alternative. During the no-visitation policy period, remote visitation was considered for terminal patients, critically ill patients, or those requiring prolonged ICU stays. Families could express their wishes for remote visitation to the healthcare team, and these requests were taken into consideration, along with the patient's clinical condition and psychological support needs, when making implementation decisions. When provided, families visited the hospital but could not enter patient rooms; instead, they were directed to a conference room where they conducted approximately 10-minute video calls with patients using hospital-provided mobile phones. The study was approved by the Ethics Committee of Kobe University Hospital (approval number: B230244). The study was conducted in accordance with the principles of the Declaration of Helsinki (1964 and its subsequent amendments). As this was a retrospective observational study using de-identified data from routine clinical care, the Ethics Committee waived the requirement for individual informed consent. The study was conducted with an opt-out approach, where patients or families could request exclusion from the study if desired.

Participants

The study included adult patients (≥18 years) admitted to the ICU who received mechanical ventilation for at least 48 hours. Exclusion criteria were as follows: (1) patients who declined participation and (2) patients who remained comatose (Richmond Agitation Sedation Scale score ≤-4) throughout their ICU stay. Patients were divided into pre-policy (February 2019-February 2020) and post-policy (April 2020-October 2022) groups, with March 2020 data excluded as a transition period.

Variables

Primary outcomes were delirium incidence (percentage of patients with the Intensive Care Delirium Screening Checklist (ICDSC) score ≥4 at least once during ICU stay) and delirium duration (number of days with ICDSC score ≥4). The main exposure variable was the no-visitation policy (pre/post), with remote visitation implementation (yes/no) as a secondary exposure variable for subgroup analysis.

Potential confounders collected included age, sex, Acute Physiology and Chronic Health Disease Classification System II (APACHE II) score, history of dementia, ICU admission after emergency surgery, benzodiazepine use, mechanical circulatory support use, and mechanical ventilation duration.

Data collection and measurement

Data were collected from electronic medical records, including demographic characteristics, clinical variables, and medication use. Delirium was assessed using the ICDSC, with a score ≥ 4 defined as delirium positive [17]. The ICDSC was administered by trained nurses every eight hours as part of routine clinical practice. The Japanese version of ICDSC has demonstrated reliability (inter-rater reliability κ=0.71) and validity (sensitivity: 80%, specificity: 74.6%) [17].

Bias

To minimize measurement bias, delirium assessment was conducted using a standardized method with the ICDSC, and all nurses received appropriate training before use. This training was part of our routine clinical practice to ensure consistent delirium screening quality, not specifically implemented for research purposes. To address potential selection bias, all eligible patients during the study period were consecutively screened, and the number of patients excluded with reasons was documented. To address confounding bias, multivariate analysis was performed with adjustment for potential confounding factors.

Sample size

The sample size for this study was determined a priori to detect a statistically significant difference in delirium incidence. Based on a review of prior literature [2,3,6] on delirium in long-term mechanically ventilated patients, we assumed a delirium incidence of 45% in the pre-policy group and aimed to detect a 15% absolute reduction (i.e., to 30%) in the post-policy group. With these assumptions, a sample size of 359 patients (183 in the pre-policy group and 176 in the post-policy group) was calculated to achieve a statistical power of 0.87 at an α level of 0.05. This calculation was performed during the study design phase and was not a post-hoc analysis based on the results.

Statistical analysis

For comparison of patient characteristics, t-tests were used for normally distributed data, Mann-Whitney U tests for non-normally distributed data, and chi-square tests or Fisher's exact tests for categorical data. To evaluate the association between delirium occurrence and the no-visitation policy, univariate and multivariate logistic regression analyses were performed. Covariates for multivariate analysis were pre-defined based on previous research and clinical expert knowledge. Age [18-20], sex, APACHE II score [18], history of dementia [21], emergency ICU admission [19,20], use of benzodiazepines [18,20,22], use of mechanical circulatory support devices, duration of mechanical ventilation [23], and no-visitation policy were selected as covariates. These factors were chosen as predictors of delirium based on previous studies. Additionally, in the post-policy group, subgroup analysis was performed to evaluate differences in delirium incidence rates and delirium duration according to the presence or absence of remote visitation. All statistical analyses were performed using R (version 4.3.2; R Development Core Team, Vienna, Austria), and p < 0.05 was considered statistically significant.

Missing data

As missing values for key variables were less than 5%, complete case analysis was performed.

## Results

Participant characteristics

**Figure 1:**
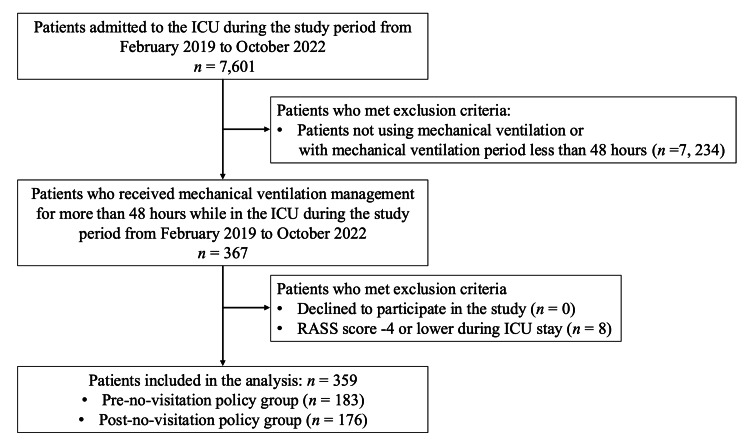
Patient selection flow diagram. Abbreviations: RASS = Richmond Agitation-Sedation Scale

During the study period (February 2019-October 2022), 367 patients received mechanical ventilation for at least 48 hours while in the ICU. After excluding eight patients who met the exclusion criteria (Richmond Agitation-Sedation Scale (RASS) score ≤-4 throughout the ICU stay), 359 patients (pre-policy: 183, post-policy: 176) were included in the final analysis (Figure 1). Complete follow-up was achieved for all study participants, with no missing data for the primary outcomes.

Table 1 shows the baseline characteristics of both groups. The median age was 69 years (IQR: 56-78), with 61.6% of patients aged ≥ 65 years. Males comprised 69.4% of the sample, with a significantly higher proportion of females in the post-policy group (38.6% vs. 28.4%, p=0.04). The median APACHE II score, indicating illness severity, was 21 (IQR: 16-27) and was significantly higher in the post-policy group (23 (17-28) vs. 19 (14-24), p<0.01). The distribution of admission diagnoses did not differ significantly between groups, with respiratory failure accounting for 73.5% of all admissions. The median duration of mechanical ventilation was eight days (IQR: 5-17) with no significant difference between groups (9 (5-19) vs. 8 (5-16), p=0.20). Regarding medication use, opioid use was significantly higher in the pre-policy group (97.8% vs. 92.6%, p=0.02), while benzodiazepine use showed no significant difference (18.6% vs. 26.1%, p=0.09).

**Table 1:** Patient characteristics pre- and post-implementation of visitation restrictions. Data are presented as median (interquartile range) for continuous variables and number (percentage) for categorical variables. For comparison of patient characteristics between groups, Mann-Whitney U tests were used for continuous data and chi-square tests or Fisher's exact tests for categorical data. Statistical significance was set at p<0.05. †Circulatory support devices include IABP, ECMO, VAD, and IMPELLA. Abbreviations: IQR = interquartile range; BMI = body mass index; APACHE = Acute Physiology and Chronic Health Evaluation; SOFA = Sequential Organ Failure Assessment; CRRT = continuous renal replacement therapy; IABP = intra-aortic balloon pump; ECMO = extracorporeal membrane oxygenation; VAD = ventricular assist device

Characteristic		Total (N = 359)	Pre-visitation policy (N = 183)	Post no-visitation policy (N = 176)	p-value
Age, years, median (IQR)		69 (56–78)	69 (56–76)	71 (55–79)	0.18
Age ≥ 65 years, N (%)		221 (61.6)	112 (61.2)	109 (61.9)	0.89
Female, N (%)		120 (33.4)	52 (28.4)	68 (38.6)	0.04
BMI, median (IQR)		22 (19–25)	23 (19–25)	22 (19–25)	0.74
Medical history	Dementia, N (%)	11 (3.1)	5 (2.7)	6 (3.4)	0.71
	Psychiatric disorders, N (%)	9 (2.5)	7 (3.8)	2 (1.1)	0.10
Clinical characteristics	APACHE II score, median (IQR)	21 (16–27)	19 (14–24)	23 (17–28)	<0.01
	SOFA score, median (IQR)	9 (7–12)	9 (7–12)	10 (7–12)	0.79
	Emergency admission, N (%)	286 (79.7)	142 (77.6)	144 (81.8)	0.32
ICU admission reason	Respiratory failure, N (%)	264 (73.5)	138 (75.4)	126 (71.6)	0.41
	Sepsis, N (%)	323 (90.0)	162 (88.5)	161 (91.5)	0.35
Interventions and outcomes	Mechanical ventilation duration, days, median (IQR)	8 (5–17)	9 (5–19)	8 (5–16)	0.20
	CRRT, N (%)	110 (30.6)	64 (35.0)	46 (26.1)	0.07
	CRRT duration, days, median (IQR)	13 (6–20)	13 (7–19)	12 (4–27)	0.72
	Circulatory support devices†, N (%)	55 (15.3)	30 (16.4)	25 (14.2)	0.56
	ICU length of stay, days, median (IQR)	13 (8–25)	13 (8–22)	13 (9–27)	0.30
	Hospital length of stay, days, median (IQR)	47 (29–84)	51 (32–87)	41 (25–80)	0.09
	ICU mortality, N (%)	52 (14.5)	32 (17.5)	20 (11.4)	0.10
Delirium and medication	Delirium incidence, N (%)	249 (69.4)	133 (72.7)	116 (65.9)	0.16
	Delirium duration, days, median (IQR)	4 (2–7)	4 (2–7)	4 (2–7)	0.75
	Opioid use, N (%)	342 (95.3)	179 (97.8)	163 (92.6)	0.02
	Benzodiazepine use, N (%)	80 (22.3)	34 (18.6)	46 (26.1)	0.09
	Remote visitation, N (%)	46 (12.8)	1 (0.5)	45 (25.6)	<0.001

Primary outcome analysis

Following the baseline comparisons, we examined our primary outcomes of delirium incidence and duration. Delirium incidence was 72.7% (133/183) in the pre-policy group and 65.9% (116/176) in the post-policy group, with no statistically significant difference (p=0.164). The median delirium duration was four (two to seven) days in both groups (p=0.593).

Multivariate analysis

In univariate analysis, the no-visitation policy was not significantly associated with delirium occurrence (OR: 0.73, 95% CI: 0.47-1.13, p=0.164). Multivariate logistic regression showed that age (adjusted OR: 1.02, 95% CI: 1.01-1.03, p=0.007), APACHE II score (OR: 0.96, 95% CI: 0.93-0.99, p=0.008), and mechanical ventilation duration (OR: 1.03, 95% CI: 1.01-1.06, p=0.003) were significantly associated with delirium occurrence. The no-visitation policy remained non-significant (OR: 0.77, 95% CI: 0.48-1.25, p=0.294) (Table 2).

**Table 2:** Univariate and multivariate logistic regression analysis for factors associated with delirium development. Univariate logistic regression analyses were initially performed with delirium occurrence as the outcome variable. Variables showing p<0.05 in univariate analysis, along with clinically important confounders, were subsequently entered into a multivariate logistic regression model. Results are expressed as odds ratios (OR) with 95% confidence intervals (CI). Statistical significance was defined as p<0.05. †Circulatory support devices include IABP, VAD, and IMPELLA. Abbreviations: OR = odds ratio; CI = confidence interval; APACHE = Acute Physiology and Chronic Health Evaluation

Variable	Univariate Analysis OR (95% CI)	p-value	Multivariate Analysis OR (95% CI)	p-value
Age (per year)	1.02 (1.00–1.03)	0.027	1.02 (1.01–1.03)	0.007
Female sex	1.05 (0.65–1.69)	0.924	1.03 (0.61–1.71)	0.924
Dementia	4.56 (0.58–36.07)	0.311	3.01 (0.36–25.31)	0.311
APACHE II score	0.97 (0.95–1.00)	0.008	0.96 (0.93–0.99)	0.008
Emergency admission	0.89 (0.51–1.57)	0.697	1.00 (0.53–1.87)	0.997
Benzodiazepine use	1.04 (0.60–1.79)	0.888	1.25 (0.70–2.25)	0.451
Circulatory support devices†	1.21 (0.64–2.30)	0.556	0.99 (0.50–1.97)	0.985
Mechanical ventilation duration (per day)	1.03 (1.01–1.05)	0.006	1.04 (1.01–1.06)	0.003
Post-no visitation policy period	0.73 (0.46–1.14)	0.165	0.77 (0.48–1.25)	0.294

Subgroup analysis: impact of remote visitation

In the post-policy group, patients receiving remote visitation (n=45) had a higher rate of emergency admissions (93.3% vs. 77.9%, p=0.02) and longer ICU stays (19 (11-38) days vs. 12 (8-25) days, p<0.01) compared to those without remote visitation (n=131) (Table 3).

**Table 3:** Characteristics of patients with and without remote visitation in the post-no visitation policy group. Data are presented as median (interquartile range) for continuous variables and number (percentage) for categorical variables. For comparison of patient characteristics between groups, Mann-Whitney U tests were used for continuous data and chi-square tests or Fisher's exact tests for categorical data. Statistical significance was set at p<0.05. †Circulatory support devices include IABP, VAD, and IMPELLA. Abbreviations: IQR = interquartile range; BMI = body mass index; APACHE = Acute Physiology and Chronic Health Evaluation; SOFA = Sequential Organ Failure Assessment; CHF = congestive heart failure; ACS = acute coronary syndrome; CRRT = continuous renal replacement therapy; IABP = intra-aortic balloon pump; VAD = ventricular assist device

Characteristic		Total (N = 176)	Remote visitation (N = 45)	No Remote visitation (N = 131)	p-value
Age, years, median (IQR)		71 (55–79)	71 (53–82)	70 (55–79)	0.72
Age ≥ 65 years, N (%)		109 (61.9)	27 (60.0)	82 (62.9)	0.76
Female, N (%)		68 (38.6)	23 (51.1)	45 (34.4)	0.05
BMI, median (IQR)		22 (19–25)	21.7 (19–25)	22 (20–25)	0.99
Medical history	Dementia, N (%)	6 (3.4)	1 (2.2)	5 (3.8)	0.61
	Psychiatric disorders, N (%)	2 (1.1)	0 (0.0)	2 (1.5)	0.40
Clinical characteristics	APACHE II score, median (IQR)	23 (17–28)	24 (19–30)	22 (16–27)	0.12
	SOFA score, median (IQR)	10 (7–12)	9 (7–12)	11 (7–13)	0.19
	Emergency admission, N (%)	144 (81.8)	42 (93.3)	102 (77.9)	0.02
ICU admission reason	Respiratory failure, N (%)	126 (71.6)	29 (64.4)	97 (74.0)	0.22
	Sepsis, N (%)	161 (91.5)	37 (82.2)	124 (94.7)	0.01
Interventions and outcomes	Mechanical ventilation duration, days, median (IQR)	8 (5–16)	12 (6–21)	7 (4–14)	0.01
	CRRT, N (%)	46 (26.1)	13 (28.9)	33 (25.2)	0.63
	CRRT duration, days, median (IQR)	12 (4–27)	14 (6–20)	9 (4–27)	0.86
	Circulatory support devices†, N (%)	25 (14.2)	13 (28.9)	12 (9.2)	<0.01
	Circulatory support duration, days, median (IQR)	4 (2–11)	7 (4–15)	3 (2–7)	0.03
	ICU length of stay, days, median (IQR)	13 (9–27)	19 (11–38)	12 (8–25)	<0.01
	Hospital length of stay, days, median (IQR)	41 (25–80)	51 (33–100)	39 (24–78)	0.08
	ICU mortality, N (%)	20 (11.4)	5 (11.1)	15 (11.5)	0.95
Delirium and medication	Delirium incidence, N (%)	116 (65.9)	29 (64.4)	87 (66.4)	0.81
	Delirium duration, days, median (IQR)	4 (27)	6 (4–12)	3 (2–6)	<0.01
	Opioid use, N (%)	163 (92.6)	42 (93.3)	121 (92.4)	0.83
	Benzodiazepine use, N (%)	46 (26.1)	14 (31.1)	32 (24.4)	0.38

No significant difference in delirium incidence was observed between patients with and without remote visitation (64.4% vs. 66.4%, p=0.81). Although delirium duration was significantly longer in patients receiving remote visitation (6 (4-12) days vs. 3 (2-6) days, p<0.01), after adjustment for illness severity, admission type, and mechanical ventilation duration, remote visitation was not significantly associated with either delirium incidence (OR: 0.94, 95% CI: 0.46-1.93, p=0.87) or duration (adjusted coefficient: 1.21, 95% CI: -0.55 to 2.97, p=0.18) (Table 4).

**Table 4:** Factors associated with delirium development to remote visitation in the post-no visitation policy group. Univariate logistic regression analyses were initially performed with delirium occurrence as the outcome variable. Variables showing p<0.05 in univariate analysis, along with clinically important confounders, were subsequently entered into a multivariate logistic regression model. Results are expressed as odds ratios (OR) with 95% confidence intervals (CI). Statistical significance was defined as p<0.05. Abbreviations: OR = odds ratio; CI = confidence interval; APACHE = Acute Physiology and Chronic Health Evaluation

Variable	Univariate Analysis OR (95% CI)	p-value	Multivariate Analysis OR (95% CI)	p-value
Age (per year)	1.00 (0.99–1.03)	0.227	1.02 (1.00–1.04)	0.095
Female sex	1.41 (0.74–2.74)	0.300	1.21 (0.61–2.43)	0.580
APACHE II score	0.97 (0.94–1.01)	0.209	0.96 (0.92–1.00)	0.083
Mechanical ventilation duration (per day)	1.03 (1.00–1.06)	0.060	1.03 (1.01-1.07)	0.042
Remote visitation	0.92 (0.45–1.89)	0.810	0.84 (0.39–1.82)	0.647

## Discussion

In this retrospective observational study, we examined the impact of COVID-19-related no-visitation policy on delirium development in ICU patients requiring mechanical ventilation for more than 48 hours, using electronic health records. The main finding was that the no-visitation policy did not significantly affect delirium incidence or duration. Additionally, remote visitation implemented after the no-visitation policy also had no significant impact on delirium incidence or duration.

Impact on delirium

Family visits and flexible visitation hours are expected to reduce the incidence and duration of delirium in ICU patients, but no such association was found in this study. Three possible mechanisms may explain these results. First, the patient population in this study - patients requiring mechanical ventilation for more than 48 hours - represents a subgroup with inherently high risk of delirium due to severe illness, prolonged sedation, immobility, and communication barriers. In such high-risk populations, the effect of family presence may be offset by more powerful delirium-inducing factors (severity, sedation, immobility, etc.). Zaal et al. [24] similarly noted that, for patients with multiple delirium risk factors, a single intervention might not significantly affect the overall incidence.

Second, the pre-pandemic visitation policy was already relatively limited (once daily for 30 minutes), which may have been insufficient to provide continuous support for delirium prevention. An ICU visit trial [16] similarly reported that flexible family visitation did not significantly reduce delirium incidence, suggesting that optimal delirium prevention likely requires multi-component interventions beyond family visits alone. The optimal strategy for delirium prevention in ICUs is likely a multi-component intervention that includes not only family visits but also early mobilization programs and environmental adjustments [25,26].

Third, enhanced non-pharmacological delirium prevention strategies by nurses may have offset the impact of family absence. During the pandemic, multi-component delirium prevention bundles including early mobilization, sleep promotion, careful sedation management, and regular reorientation were strengthened. A meta-analysis by Fang et al. [27] of 18 studies involving 2,717 participants demonstrated that these care bundles significantly reduced delirium incidence (RR=0.38, 95% CI: 0.32-0.45; p<0.001) and delirium duration (WMD = -1.60, 95% CI: -1.96 to 1.23; p<0.001). This meta-analysis highlights the importance of a comprehensive approach, including psychological support, environmental adjustment, appropriate medication management, early rehabilitation, and complication prevention, suggesting that strengthening these nursing interventions is essential, especially when family visits are restricted.

Regarding WMD, it is noteworthy that the no-visitation policy did not lead to prolongation. This may reflect the special circumstances during the pandemic. During the COVID-19 pandemic, while patients experienced anxiety about infection and stress from isolation, healthcare professionals may have intensified intentional psychological support and cognitive stimulation. These interventions may have complemented the absence of family visits and prevented negative effects on WMD. This suggests that appropriate nursing care can prevent worsening of delirium even in crisis situations.

Impact of remote visitation

Subgroup analysis revealed that the remote visitation group had a significantly higher proportion of severe patients (higher emergency admission rate, longer ICU stay) compared to the non-remote visitation group. This suggests that remote visitation was prioritized for patients with higher expected psychological needs for families, such as terminal patients or long-term patients. However, despite this prioritization, remote visitation had no significant impact on delirium incidence.

This may indicate limitations of digital technology as a substitute for face-to-face visits. Shahdosti et al. [28] reported reduced anxiety scores with frequent online video visits, but physical presence elements such as tactile communication and non-verbal cues may not be sufficiently reproduced virtually [29].

To maximize remote visitation effectiveness, standardized protocols regarding frequency, quality, and timing may be necessary. Nurses serving as communication facilitators between patients and families may improve remote visitation quality, and customized approaches based on patients' cognitive status and consciousness levels are important.

Limitations

Strengths of this study include its focus on high-risk patients requiring prolonged mechanical ventilation and the use of validated delirium assessment tools administered by trained ICU nurses.

Additional limitations include the retrospective design limiting causal inference, lack of detailed data on remote visitation protocols (frequency, duration, quality), potential unmeasured confounders such as changes in nursing care practices during the pandemic, and limited statistical power to detect clinically meaningful differences as revealed by post-hoc analysis. The single-center design may limit generalizability to other ICU settings with different patient populations or care protocols.

## Conclusions

In this study, no-visitation policy and remote visitation did not significantly affect the incidence or duration of delirium in ICU patients requiring long-term mechanical ventilation. This suggests that, for delirium prevention in ICUs, rather than relying solely on family visits, a multi-component approach that actively implements non-pharmacological interventions by nurses, such as early mobilization and cognitive stimulation, is important.
